# Supplementary material for: Chromothripsis during telomere crisis is independent of NHEJ, and consistent with a replicative origin
Source: Genome Res. 2019 May;29(5):737–49. doi: 10.1101/gr.240705.118 (PMC6499312; doi:10.1101/gr.240705.118)
Supplement: Supplemental Material [file supp_gr.240705.118_Supplemental_file_1.zip › contigs/annotated_contigs/DB79/contig.2.DB79_length_712_mean_cov_9.27387640449.docx]

**DB79_length_712_mean_cov_9.27387640449**

```
CATATGTGTGTTTTAACTCTAAATTAGAATATACATTAAAGAAATTACTTATCAATCCATACAAATCATGCAATATATTGCCATGTCAA
 >chr9:118696581-118696906 + E=9e-184 p=0e+00
TGTATTTGGACTTATTAGTCTCCGGAATTGCCTTTTGAGTGGTGTGGTAAACTCACTACTCTCTCTCTCACTCTCACTCATTCTCTCAC

TCATTTTCTCTCTCTCTCTCTCTCTCTCTCTCTCTCCATATATATATATATATTCCCTGCCTGATATTACATAGTACATGGAGGCAGGA

GGCAGGATGAGGAGGGGAATTAGCATGGATTTGAGACACCAGAAGACTGAACTTCAAT|TCTAGATTT|TTTCCCACCTGGTTTGCACA
                                                                    >chr9:98169904-9817
ATCTTCAGTTCTGCAGTAATCTTTTCTTTTTTTTCTTTTCTTTTCTTTCTTTCTTTCTTTTTCTTTCTTCCTCTTTTTCTTCCTTCCTT
0282 + E=1e-203
TCTTTCTTTCCTTCTTTCTTTCTCTCTCTCCTTTTCTTTCTTTCTTCTTTCTCTCTCCTCTCTTTCCTCTTTCTTTTTTTTCTCTCTCT

TTCTCTTCTTTTGTTTTTCTTTTGAGATGGAATTTCACTCTTGTGGCCCAGGCTGGAGTGCAATGGCATGATCTTGGCTCACTGCAAAC

TCTGCCTCCCAGGTTCAAGCGATTCTCCTGCCTCAGCCTCCCGAGTAGCTGGGATTACAGGCATGCACCACCATGCCAGGCTAATTTTG

TA
```
